# 

**Published:** 1913-10-04

**Authors:** 


### CONTENTS.

| | PAGE |
|---|---|
| **The British Medical Association and its Critics** | 1 |
| **Red Cross Uses of Aerial Transport** ... ... | 2 |
| **Hospital and Institutional News,** including— "The Hospital's" Special Mental Hospital Number—The Downs Sanatorium Investigations—Medical Officers and the Cost of Dispensing—A Progressive Fight by the Camberwell Guardians—A Verandah Mental Hospital—The Centralisation of Serology—The Almoner System and the Hospital Saturday Fund—Re-opening of the School of Pharmacy. | 3 |
| **The Bacteriology of Food Poisoning** ... ... | 9 |
| **The Admissions to a Voluntary Hospital** ... New Admission Block at Royal Victoria Infirmary, Newcastle. | 11 |
| **Legal Notes for Institutional Workers** ... ... | 12 |
| **Insurance Sanatorium Construction** ... ... Hints to Architects by a Sanatorium Superintendent. | 13 |
| **Buildings Contemplated and in Progress** ... | 14 |
| **The Institutional Library** ... ... ... ... ... The Prototype of Sherlock Holmes—Treatment after Operations—Incipient Phthisis, and Thermal Environment—The Pocket Guide Series. | 15 |
| **The Unsatisfactory Panel System** ... ... ... | 17 |
| **Appointments** ... ... ... ... ... ... | 18 |
| **Blind-Alley Hospital Employments** ... ... The Case of the "Lab" Boy. | 19 |
| **Institutional Needs** ... ... ... ... ... ... | 20 |
| **The Editor's Letter-Box** ... ... ... ... The Bane of Influence in Phthisis Work—Certificates for Hospital Life Governors. | 21 |
| **The National Medical Guild** ... ... ... The Lesson of the Manchester System. | 22 |
| **The Institutional Worker** ... ... (Suppl.) Gas for Hospitals—The Gas Exhibition. | 1 |
| **Our Bureau of Information** ... ... (Suppl.) | 1 |

